# Supplementary material for: The Systems Biology Research Tool: evolvable open-source software
Source: BMC Syst Biol. 2008 Jun 29;2:55. doi: 10.1186/1752-0509-2-55 (PMC2446383; doi:10.1186/1752-0509-2-55)
Supplement: Additional file 1 — SBRT Archive. An archive of the current version of the Systems Biology Research Tool. [file 1752-0509-2-55-S1.zip › sbrt-1.4.0/doc/developers_guide/api/sbrt/kernel/math/stat/comp/class-use/Correlator.html]

Uses of Interface sbrt.kernel.math.stat.comp.Correlator


|  |  |  |
| --- | --- | --- |
| **Overview** \| **Package** \| **Class** \| **Use** \| **Tree** \| **Deprecated** \| **Index** \| **Help** | |  |
| PREV   NEXT | **FRAMES**    **NO FRAMES**     **All Classes** |  |


---


## **Uses of Interface sbrt.kernel.math.stat.comp.Correlator**

| Packages that use Correlator | |
| --- | --- |
| **sbrt.ext.R.stat** | Provides classes that allow communication with R for the purposes of statistics. |
| **sbrt.kernel.math.stat.comp** | Provides classes and interfaces for performing statistical computations. |
| **sbrt.kernel.math.stat.proc** | Provides classes and interfaces for performing statistical processes. |

Uses of Correlator in sbrt.ext.R.stat

| Classes in sbrt.ext.R.stat that implement Correlator | |
| --- | --- |
| `class` | `RCorrelator<V>`             This class computes correlation statistics using `R`. |
| `class` | `RKendall<V>`             This class computes Kendall tau values using R. |
| `class` | `RPearson<V>`             This class computes Pearson r values using R. |
| `class` | `RSpearman<V>`             This class computes Spearman rho values using R. |

Uses of Correlator in sbrt.kernel.math.stat.comp

| Classes in sbrt.kernel.math.stat.comp that implement Correlator | |
| --- | --- |
| `class` | `KendallTau<V>`             This abstract class is a skeleton implementation of a Kendall's tau correlation statistic computer. |
| `class` | `KendallTauA<V>`             This class is used to compute Kendall's Tau_A_ correlation statistic. |
| `class` | `KendallTauB<V>`             This class is used to compute Kendall's Tau_B_ correlation statistic. |

Uses of Correlator in sbrt.kernel.math.stat.proc

Constructors in sbrt.kernel.math.stat.proc with parameters of type Correlator

`CorrelationAnalysis(Correlator<V,T> correlator)`
  
          Constructs a new correlation experiment.

`MultipleCorrelationAnalysis(Correlator<V,T> correlator)`
  
          Constructs a new correlation experiment.

---


|  |  |  |
| --- | --- | --- |
| **Overview** \| **Package** \| **Class** \| **Use** \| **Tree** \| **Deprecated** \| **Index** \| **Help** | |  |
| PREV   NEXT | **FRAMES**    **NO FRAMES**     **All Classes** |  |


---
